# Prevalence, temporal course and risk factors for phantom eye symptoms in uveal melanoma

**DOI:** 10.1038/s41433-023-02756-w

**Published:** 2023-09-26

**Authors:** Stephen L. Brown, Laura Hope-Stone, Rumana N. Hussain, Heinrich Heimann, Nicola van der Voort, M. Gemma Cherry

**Affiliations:** 1 School of Psychology, University of New England, Armidale, NSW Australia; 2 Department of Primary Care and Mental Health, University of Liverpool, Liverpool, UK; 3 Liverpool Ocular Oncology Centre, Liverpool University Hospital NHS Foundation Trust, Liverpool, UK

**Keywords:** Eye manifestations, Pain

## Abstract

**Background:**

Phantom eye symptoms (PES), particularly phantom visual sensations (PVS) and phantom eye pain (PEP), are common in enucleated patients and can lead to psychological distress. Current cross-sectional studies cannot examine the temporal course of symptoms, nor can they identify dynamic risk factors or consequences of PES.

**Methods:**

Cohort study of 105 enucleated uveal melanoma patients returning self-report questionnaires, within 4 weeks of diagnosis and 6-, 12- and 24-months post-treatment. Questionnaires measuring PVS and PEP symptoms in the week prior to completion, pain severity, Hospital Anxiety and Depression Scale scores and the Functional Assessment of Cancer Therapy scale (FACT-G) measuring quality of life.

**Results:**

PVS and PEP emerged after 6 months, were relatively stable over the study and did not remit. PVS showed 6-, 12- and 24-month prevalence rates of 44.6%, 48.2% and 30.2%, and PEP 16.1%, 18.4% and 17.5% respectively. PVS were generally elementary, with only 10–15% of the total cohort experiencing complex sensations. PEP was generally neither prolonged nor intense, except in a small proportion. PVS and PEP were showed moderate associations but did not predict each other prospectively. Anxiety within 4 weeks of diagnosis was a risk factor for the initiation of PEP. Neither PVS nor PEP prospectively predicted anxiety, depression or quality of life.

**Conclusions:**

PES were prevalent and non-remitting, beginning within 6 months of enucleation. PVS and PEP may not represent symptoms of a coherent syndrome. We discuss findings with reference to theories of phantom sensations, and directions for clinical practise and research.

## Introduction

Uveal melanoma (UM) is the most common intraocular tumour in adults. Approximately 30% of UM patients are treated by enucleation [1]. Enucleation is commonly experienced as traumatic, and is associated with pain, adverse physical and functional consequences, psychological distress and poorer quality of life (QoL) [2].

Additionally, UM patients may experience phantom eye symptoms (PES) [3]; pain or visual sensations that appear to emanate from the removed eye. Clinical characteristics and correlates of PES have been documented across populations of UM, eye infection and eye injury amputees [4–6]. Phantom visual sensations (PVS) are reported by 30–42% of patients; including unstructured phenomena, such as colours or shapes, and, less frequently, meaningful images such as faces or animals. About a quarter of patients report ‘seeing’ from the absent eye [3]. Phantom eye pain (PEP) is reported by 23–47%, and phantom tactile sensations by 2%. PES are often perceived as disturbing and are associated with elevated anxiety and depression [3, 5, 6].

PES is yet to be fully described, because previous studies are cross-sectional and identify neither the temporal course nor precursors or consequences of PES. This limits the interpretation of findings in four ways. First, PES symptom timelines, initiation, consistency and remission, are unknown. Second, it is unclear how symptoms are temporally linked. Initially PES was considered a unique syndrome [2], but PVS and PEP are not strongly linked in cross-section [3, 5]. It is unclear, though, whether PVS and PEP form part of a syndrome with links at some timepoints and divergences at others, or whether PVS and PEP are sequentially linked such that one precedes the other.

Third, PEP is associated with pre-amputation eye pain [3, 5]. In the phantom limb pain literature, such associations are cited as evidence of maladaptive neural plasticity [7] or prior learning [8]. However, cross-sectional studies assess pre-amputation pain simultaneously with PEP measures, overlooking the possibility that recall of prior pain may be influenced by post-amputation PEP. Fourth, PES, particularly PEP, are cross-sectionally associated with elevated anxiety and depression [3, 5, 6]. The direction of this association is unknown. Psychological distress may cause, phantom limb pain [9]. Equally plausibly, pain is a well-established cause of psychological distress [10]. PES are subjectively disturbing [2, 3] and may cause anxiety and depression.

To overcome the problems of cross-sectional studies, we conducted a two-year prospective cohort study of UM patients. We aimed to identify the putative course, precursors and consequences of three major PES symptoms; PVS, ‘seeing’ and PEP. Our first aim was to establish symptom prevalence at 6, 12 and 24 months post-diagnosis, and intra-individual symptom consistency. Our second aim was to establish whether PES are prospectively predicted by other PES symptoms, pain, anxiety, depression or QoL. Our third aim was to determine whether PVS and PEP prospectively predict anxiety, depression or QoL.

## Methods

### Ethical oversight

The conduct of the study was approved by the Liverpool Central NHS Ethics Committee (03/06/072/A).

### Study design

Open cohort study with return-paid questionnaires administered at baseline (within 4 weeks of diagnosis), and 6-, 12- and 24-months later. Anxiety and depression were measured at all timepoints and PVS, PEP and QoL at 6-, 12- and 24-months. Clinical and demographic variables were controlled.

### Participants

We recruited consecutive adult UM patients treated by enucleation at the Liverpool Ocular Oncology Centre (LOOC) for UM (choroid and ciliary body) tumours between July 2015 and July 2020. At LOOC, patients are treated by enucleation if eye-conserving procedures are not clinically indicated or by patient preference [11]. All patients who gave written consent for this study were posted the self-report questionnaire with enclosed postage-paid envelopes 4 weeks 6, 12 and 24 months following diagnosis. Analyses were confined to participants who returned questionnaires at two or more timepoints.

### Measures

Socio-demographic and clinical data were available from clinical records for all participants. Clinical data included affected eye, visual acuity, tumour diameter, extra-ocular extension and prognostic testing outcomes. Visual tests used the Snellen method converted to logMAR scores. Extra-ocular extension is tumours that extend beyond the eye and are often treated by external beam radiotherapy to the socket following enucleation. Prognostic testing outcomes were also included. About 40% to 50% of UM patients will develop metastatic disease within 10 years, for which treatment rarely pro-longs life [12]. Metastatic risk and all-cause mortality are predicted by multiple clinical, histological and tumoural genetic risk factors; the strongest predictive factor involves a mutation deletion of one of the pair of chromosome 3 alleles (Monosomy 3-M3) [13]. LOOC offers prognostic testing with outcomes communicated within six weeks. Testing outcomes include M3, Disomy 3 (D3 - absence of mutation) or unknown (patient did not accept testing offer or test failed).

PVS and PES were measured using questionnaire items derived from those of Rasmussen et al. [6], Martel et al. [5] and Hope-Stone et al. [3]. To normalise what some may see as unusual or discomforting sensations^4^, we provided the following statement on the questionnaire; ‘Some people experience visual sensations and pain that feel as though they come from the removed eye’. Participants were then asked to report if they experienced ‘visual sensations in the removed eye’ during the past week. If so, they were asked if these sensations resembled the following; visual patterns, flashing lights, shapes, kaleidoscopes, colours, people, animals or other (taken from Hope-Stone et al. [3]). Participants were then asked to report if they felt that they could ‘see through the removed eye’ during the past week (yes/no), and an open text question concerning what they ‘saw’. Participants were asked if they had experienced pain in the last week (yes/no), and if so for what duration (few minutes/few hours/few days/whole week). They were also asked to indicate the worst pain during that week, on a 1–10 scale anchored by the terms ‘no pain’ and ‘as bad as you can imagine’. Previous studies have found pain to be associated with reports of pre-surgical pain in the eye. Thus, we asked participants if they had experienced pain in the eye before removal (yes/no).

Anxiety and depression were assessed using subscales of the Hospital Anxiety and Depression Scale (HADS) [14]. Each has seven items scored from 0 to 3 with higher scores signifying greater symptomology (range = 0–21). Both subscales predict diagnosed cases with good sensitivity and specificity [15].

QoL was measured using the total score from the Functional Assessment of Cancer Therapy scale (FACT-G) [16]. The FACT-G is a 28 item, five-point scale from 0 (not at all) to 4 (very much). Item scores are summed with a range of 0–108, with higher scores indicating better QoL.

### Analysis

#### Aim 1

Temporal courses of PVS and PEP were inferred from prevalence rates at each time-point. We examined consistency of symptom reporting by calculating percentages of participants reporting symptoms across timepoints.

#### Aim 2

Using binary logistic regressions, PVS and PEP outcomes at 12 and 24 months were regressed onto PEP, PVS and QoL measured at 6 and 12 months respectively, and from anxiety and depression at each timepoint. As shown in Table 1, ‘seeing’ was uncommon and all patients who reported ‘seeing’ also reported PVS. Thus, we did not conduct separate analyses to predict ‘seeing’. Initial values of the outcome variable were controlled. Odds ratios and 95% upper and lower confidence intervals (CIs) are reported.

**Table 1:** Prevalence Rates of Phantom Eye Symptoms and Mean (SD) Anxiety, Depression and Quality of Life at Each Timepoint.

	Pre-Treatment (*n* = 86)	6 Months (*n* = 89)	12 Months (*n* = 93)	24 Months (*n* = 64)
Visual Sensations		37 (44.6%)	44 (48.2%)	19 (30.2%)
Visual patterns		5	7	0
Flashing lights		12	15	7
Shapes		13	14	7
Kaleidoscopes		14	22	9
Colours		7	6	1
People		3	5	3
Animals		1	2	1
Other		8	5	3
‘Seeing’ real things		10 (11.2%)	11 (11.8%)	6 (9.4%)
Pain		14 (16.1%)	16 (18.4%)	11 (17.5%)
Time in Pain - minutes		7	10	5
Hours		3	2	2
Days		1	1	2
Weeks		2	3	1
Pre-amputation eye pain		13 (15.3%)	15 (17.0%)	9 (14.3%)
Pain Intensity		0.86 (SD = 1.61)	1.15 (2.09)	1.26 (2.06)
Anxiety	6.27 (SD4.65)	5.63 (SD4.71)	5.51 (SD4.64)	5.80 (SD4.08)
Depression	3.87 (SD4.01)	4.47 (SD4.78)	3.94 (SD4.09)	4.68 (SD5.06)
QoL		63.70 (SD14.68)	61.75 (SD13.10)	58.41 (SD8.54)

#### Aim 3

We used linear regression, to predict 12- and 24-month anxiety and depression from PVS and PEP at 6 and 12 months.

#### Statistical controls

To eliminate confounding, we examined the association between demographic and clinical variables and PVS and PEP. None predicted PVS or PEP, and thus none were used as control variables.

#### Missing data

Aim 1 analyses used returned data with no data replacement. For Aim 2 analyses, missing data were replaced by multiple imputations. Ten imputations were used to replace missing data. Eleven participants died during the study, 2 between 6 and 12 months and none between 12 and 24 months. Missing data due to death was addressed by creating a covariate representing time points (from 2–4) for which patients were alive for imputation, we then deleted all imputations made after death [17].

## Results

During the recruitment period, 224 patients were enucleated, with 121 returning at least one questionnaire. Of the 121, 105 met the criterion of having returned questionnaires at two or more time points. Demographic and clinical data were available for all 105 patients. The baseline questionnaire was returned by 86 (81.9%), 6-month return was 89 (84.8%), 12-month return was 93 (88.6%, two patients died before this time point) and the 24-month return was 64 (61.0%, another nine patients died before this time point). Attrition analysis showed that a 24-month dropout (not attributable to death) was not predicted by any study variable. Table 2 shows distributions of demographic and clinical characteristics.

**Table 2:** Socio-Demographic and Clinical Characteristics of Participants.

	Number/Mean	Percentage/*SD*
Age	Mean = 70.95	SD = 11.46
Sex		
Males	53	50.5
Females	52	49.5
Marital Status		
Married or With Partner	80	76.2
Widowed	7	6.7
Divorced/Separated	8	7.6
Single	10	9.5
Employment		
Employed	28	26.7
Not Employed	2	1.9
Retired	63	60.0
Illness leave from employment	3	2.9
Other	6	8.6
Eye		
Left	56	53.3
Right	49	46.7
Visual acuity affected eye (logMAR)	Mean = 0.356	SD = 0.426
Visual acuity fellow eye (logMAR)	Mean = 0.283	SD = 0.373
Tumour diameter (mm)	Mean = 15.64	SD = 3.80
Extra-ocular extension	10	11.4
Chromosone 3 status		
Monosomy 3 (M3)	55	52.4
Disomy 3 (D3)	25	23.8
Unknown	25	23.9

### Aim 1: PES emergence, temporal course and consistency over time

#### Phantom visual sensations

Table 1 shows consistent prevalence rates across timepoints, with symptoms commencing before 6 months, and a non-significant reduction between 12 and 24 months. PVS were mainly elemental perceptions of flashing lights, shapes or kaleidoscopes, with a minority perceiving meaningful images of people or animals. Of 96 participants responding to the PVS item at two or more time points, 25 (26.04%) reported visual sensations at all time points (13 at three time points, 7 at two time points), and 38 (39.6%) reported visual sensations at some time points but not others. Thirty-three did not report visual sensations at all (23 at three time points, 10 at two time points). No participant who reported PVS after six months showed spontaneous remission, defined as no PVS during the subsequent two time points.

#### ‘Seeing’

About 10–12% of participants reported ‘seeing’ from the eye. Open text responses were evenly split between elementary or complex images similar to PVS (all of whom also reported PVS) and a non-specific feeling ‘as though the eye was still there’. As the former category fully overlapped PVS, we included these in that category. This left 3–5 participants who felt as though the eye existed but did not report specific visual sensations. This category was too small for meaningful analyses.

#### Pain

PES prevalence rates were consistent across time points but lower than PVS; 16.1% at 6 months, 18.4% at 12 months, and 17.5% at 24 months. The modal experience of pain lasted only minutes and mean pain intensity was generally low at just over one on the 10-point scale. Spearman rank-order correlations showed pain intensity and duration to be significantly linked; 6-month *rho* = 0.50, *p* = 0.080, 12-month *rho* = 0.56, *p* < 0.01, 24-month *rho* = 0.69, *p* < 0.01. This suggests a small number of participants reporting pain that was both more prolonged and of higher intensity that others in the sample. Of the 101 who responded to the PEP item at two or more time points, 8 (7.9%) reported pain at each time point (2 at three time points and 6 at two time points) and 35 (34.7%) reported pain at some time points and not others. 58 reported no pain at any time point (39 at three time points, 19 at two time points). No participant reported spontaneous remission.

Visual sensations and pain were moderately cross-sectionally linked at each timepoint: Timepoint 1, Φ = 0.24, *p* < 0.05; Timepoint 2, Φ = 0.33, *p* < 0.01; Timepoint 3, Φ = 0.33, *p* < 0.05.

Prevalence estimates were unlikely to be biased by the high dropout between 12 and 24 months (Table 1). In logistic regression analyses predicting retention at 24 months, neither 12-month PVS (Odds ratio = 1.24, 95% CI = 0.44, 3.133) nor PEP (Odds ratio = 1.35, 95% CI = 0.37, 4.88) predicted retention.

### Aim 2: prospective predictors of PVS and PEP

Prior observations of PEP and did not predict PVS and prior observations of PVS did not predict PEP (Table 3). Thus, PVS and PEP were not sequentially linked. PEP was not predicted by pre-amputation eye pain. The initiation of PEP at 6 months was predicted by higher levels of baseline anxiety but not baseline depression (see Table 3). Neither PVS nor PEP were predicted by anxiety, depression or QoL at any time points subsequent to 6 months.

**Table 3:** Odds Ratios with 95% Confidence Intervals for Predictors of Visual Sensations and Pain.

	Predicting 6-month from Baseline	Predicting 12 month from 6 month	Predicting 24 month from 12 month
Visual sensation			
Prior Visual Sensation		1.60 4.55 12.96	2.10 7.12 24.14
Pain		0.20 0.88 3.93	0.27 1.24 5.75
Anxiety	0.93 1.04 1.17	0.95 1.06 1.19	0.91 1.04 1.19
Depression	0.90 1.05 1.22	0.90 1.03 1.19	0.87 1.05 1.26
QoL		0.96 1.01 1.07	0.96 1.05 1.15
Pain			
Prior Pain		3.10 12.83 53.29	4.11 23.00 128.68
Visual Sensation		0.62 2.87 13.31	0.57 3.67 23.45
Anxiety	1.00 1.18 1.38	0.93 1.09 1.28	0.96 1.14 1.35
Depression	0.98 1.18 1.41	0.90 1.04 1.22	0.90 1.07 1.28
QoL		0.96 1.01 1.07	0.95 1.02 1.08
Pre-amputation eye pain	0.65 0.91 2.31	0.78 1.02 1.45	0.70 0.99 1.66

Note: Confidence intervals are displayed in smaller scripts.

### Aim 3: PVS and PEP as predictors of anxiety, depression and poorer QoL

Table 4 shows that neither PVS nor PEP predicted later depression, anxiety or poorer QoL.

**Table 4:** Unstandardised Regression Coefficients (SEs) for Predictors of Anxiety and Depression.

	Predicting 12 months from 6 month	Predicting 24 months from 12 month
Anxiety		
Prior Anxiety	0.68** (0.30)	0.32* (0.17)
Visual Sensations	0.12 (1.09)	0.82 (1.04)
Pain	2.04 (1.28)	0.68 (1.63)
Quality of Life	−0.84 (2.86)	−1.04 (3.67)
Depression		
Prior Depression	0.62** (0.58)	0.56** (0.18)
Visual Sensations	−0.32 (0.52)	0.90 (1.61)
Pain	0.77 (0.68)	0.85 (2.18)
Quality of Life	−0.36 (2.49)	0.13 (2.98)

*p < 0.05; **p < 0.01.

## Discussion

PVS and PEP were relatively common in newly enucleated UM patients, emerging before 6 months and largely persisting over 24 months. PVS were experienced by about a third to a half of participants and were generally elementary with only about a quarter of sensations consisting of meaningful images. PEP was experienced by 11–16% of participants. For most, PEP was neither prolonged nor intense, although for a small proportion of participants length of pain was related to intensity. PVS and PEP were not sequentially related. PEP was more likely to occur in patients who reported elevated pre-treatment anxiety but not elevated depression or poorer QoL. Anxiety probably constitutes a risk factor for the emergence of PEP, but does not influence its course. We found no evidence that PVS or PEP may cause elevated anxiety, depression or poorer QoL. We discuss the importance of all findings with reference to theoretical and clinical implications.

### Phantom visual sensations

PVS prevalence of 30–48% is generally in line with previous studies [2–6], although one detected prevalence rates of up to 60% [18]. Our estimate may be slightly low because we asked participants to report only symptoms during the previous week. Previous studies imposed no truncation. Consistent with previous studies, PVS mainly consisted of elementary shapes and colours, with few complex or meaningful sensations such as people or animals [3–6]. Evidence of intra-individual consistency comes from the high likelihood that those reporting PVS did so at the previous time point, and that 26% of participants reporting sensations at all time points. Nonetheless, almost 40% of participants reported PVS at some time points and not others. Taken together, these findings suggest that PVS is episodic, sometimes abating for a week or more, but endures at least 2 years after surgery. Symptom persistence is consistent with cross-sectional studies showing PVS many years post-surgery [3, 5]. Although previous studies show that PVS can be discomforting and frightening [3–5], findings indicate that PVS probably do not cause elevated anxiety or depression symptoms or affect QoL.

### ‘Seeing’ with the amputated eye

Less than 12% of participants felt that they could ‘see’ with the removed eye, compared with 28–40% in previous studies [3, 4, 19]. Truncating symptom reporting to may reduce prevalence estimation. Qualitative data raised the question of what is meant by ‘seeing’. About half of the responses pertained to elementary or complex visual sensations similar to PVS, and all of these participants also reported PVS. Others described non-specific ‘seeing’ with both eyes. The meaning of this is unclear. Participants may have experienced a form of sensory embodiment, whereby fellow eye vision is perceived to derive from the amputated eye [20].

### Phantom pain

The 16–18% prevalence rate of patients who reported PEP was lower than previous literature [3, 5, 6]. Again, this may be attributable to limiting the reporting period to the past week. Prevalences of PEP were lower than PVS, but again reasonably stable over the study. Similar to PVS, about 35% of participants reported PEP at some but not other time points, suggesting that PEP is also episodic. Fortunately, episodes were generally brief and pain mild. About 75% of participants experienced PEP for minutes or hours rather than days or weeks, and mean ratings were about one point on a ten-point scale. Nonetheless, strong associations between PEP duration and intensity existed in a small number of participants. It is unclear whether their prolonged and intense PEP merely quantitatively differs to others, or whether it had a separate aetiology. This could be a focus of future research. Also, we cannot be sure that pain represents true PEP, as we did not examine socket or adjacent structures for causes of pain.

Anxiety preceded PEP, and may cause its initiation. There was no evidence that anxiety was prospectively associated with PEP after 6 months, thus anxiety may initiate rather than maintain PEP. Depression showed a similar link although this was not statistically significant. Elevated anxiety may be a risk factor for PEP. It is unclear whether the heightened anxiety that precedes PEP is attributable to UM, or whether participants were anxious before diagnosis. Contrary to previous studies [3, 5, 6], we did not find that pre-operative eye pain predicted PEP at any time point. We did not find evidence suggesting that pain causes elevated anxiety, depression or reduced QoL.

### Is PES a syndrome?

The term ‘syndrome’ implies common cause of symptoms, with a strong empirical association between them. Similar to previous studies [3, 4], PEP and PVS were not strongly cross-sectionally related. Prospectively, we found no evidence of sequencing (e.g., one preceding the other) as part of any developmental pathway. As neither cross-sectional nor longitudinal studies have found PVS and PEP to be strongly linked, we find it unlikely that a common pathological process underlies PVS and PEP.

### Strengths and limitations

This study is the first to directly observe the temporal development of PES and make a prospective assessment of risk factors. The study also benefits from a homogenous group of participants, enucleated UM patients, who are at higher risk of PES than other enucleated patients [5]. We asked patients only to report symptoms occurring during the past week to minimize recall error, but this bracketing of the recall period can lead to prevalence underestimation.

The main drawback of the study is the lack of power afforded by 105 participants. This is particularly important because change is modeled through statistical control of the autocorrelation. Although providing a rigorous test of sequence, controlling the autocorrelation reduces power. A further problem is that 105 of 224 eligible patients were recruited, which may cause unidentified sampling bias. We also note the temporal limitation of being unable to describe trends beyond the two years of the study. We did not conduct socket examinations, and thus cannot definitively eliminate anatomical explanations for PES. Nor did we eliminate sub-clinical phenomena at the local level (e.g., neuroma of the optic nerve) or discomfort related to prostheses, and thus acknowledge limitations of any interpretations based on broader neural systems. Caution in generalising findings to other eye amputation patients is advised due to the unique demographic, medical, and psychological characteristics of both UM patients and patients undergoing enucleation. In relation to the latter, we note that previous studies have not found consistent differences between enucleated and eviscerated patients in PES prevalence or characteristics [2, 5, 6, 10].

### Theoretical implications

A number of theoretical explanations for phantom sensations have been proposed, most derived from the phantom limb and medically unexplained pain literature [9]. Probably the most relevant to our findings are constructivist theories that attribute symptom experience to the ways in which past experience influences the perception of afferent sensation [20, 21]. Neural systems are viewed as trained units that continually generate, test, and refine hypotheses about afferent inputs. For example, neuromatrix theories [20] describe perception as deriving from the processing of afferent sensory input through sub-systems relating to temporospatial positioning, emotional excitation and cognition. The neural matrix is trained in the sense that sub-systems are sharpened and differentiated in a neural system where the amputated body part existed [20, 21]. Systems are resilient to disruption because the matrix ‘fills in’ for the absence of expected, but no longer available, sensory inputs by reconstructing those inputs. ‘Filling in’ is seen to create phantom sensations [22].

These theories help to elucidate two of our findings. PVS and PEP may not be strongly related because visual and pain sensations involve differing neural systems. This would also explain why PVS is more common than PEP; visual sensations are more common in normal functioning than pain sensations and thus are likely to create stronger expectations. Further, neuromatrix theories of PEP describe emotional activation as an important determinant of perception, particularly pain perception, prior to amputation [20–22]. This may explain why pre-existing anxiety constitutes a risk factor for PEP. We emphasise, though, that this link could also be explained by attentional, resource depletion, and cognitive-behavioural accounts of pain [9].

Two findings, though, are inconsistent with constructivist accounts. First, phantom sensations should decline as neural systems gradually change to accommodate the loss of afferent input [9]. We found little evidence of decline over two years. Further, cross-sectional studies examining longer post-surgical periods have not found negative associations between PVS, PEP and time elapsed since treatment [3, 5]. Second, experience of pre-surgical eye pain should predict PEP, as it does with other forms of phantom pain, because this would help may train neural expectations for eye pain [7–9]. Cross-sectional studies have observed this, but our prospective study did not.

### Clinical implications

Although PES may not influence anxiety, depression or QoL, phantom sensations can be disturbing particularly when they are poorly understood by patients [3, 5, 6]. As PVS or PEP appears to lack clear risk factors, it highlights the importance of informing every patient that PES may occur, are normal and that PES are usually not harmful. A number of studies document triggers for PES and strategies that patients spontaneously use to reduce sensations [3, 6]. Whilst triggers and helpful strategies may be specific to individual patients, patients should be encouraged to try as many as possible to see what is helpful in their case. It is notable that anxiety precedes and may be causally-related to PEP. Thus, treating anxiety at 6 months may help to reduce PEP.

A small number of participants experienced intense non-transient pain, although it is possible that this pain originates from damaged extra-orbital structures which does not represent phantom pain. Early research in other areas of PEP is starting to show the efficacy of surgical and behavioural strategies that reduce pain through therapies such as mirroring [23], transcranial magnetic stimulation [24] and sensory feedback [25]. These are not currently directed toward PES, and will have to be adapted because mirroring and feedback techniques are primarily visual. Nonetheless, these represent a start and researchers could adapt and trial some for PES.

## Summary

### What was known before

- Phantom visual sensations and pain are known to be common in enucleated patients. Phantom sensations and pain are disturbing and are associated with anxiety and depression. Studies are cross-sectional and cannot identify the temporal course of symptoms or potential causes or consequences.

### What this study adds

- Phantom symptoms are episodic and endure at least two years after enucleation. There is little evidence that symptoms represent a coherent syndrome. Higher anxiety scores may be a risk factor for phantom pain, but there is little evidence that phantom symptoms cause anxiety, depression or reduce quality of life.

## Data Availability

Data is available from the first author subject to ethical oversight of its use.
